# Planning and Design of Low-Power-Consuming Full-Outer-Air-Intake Natural Air-Conditioning System

**DOI:** 10.1155/2019/6939632

**Published:** 2019-04-14

**Authors:** Chien-Lun Weng, Lih-Jen Kau

**Affiliations:** Department of Electronic Engineering, National Taipei University of Technology, No. 1, Sec. 3, Chung-Hsiao E. Rd., Taipei 10608, Taiwan

## Abstract

A person stays indoors for about 85%∼90% time of his lifetime, and the need for a comfortable indoor environment is getting higher; thus, the air-conditioning dependency becomes intense too. Nowadays, residents focus on both the comfortable living environment and indoor air quality. A closed environment will become hazardous because of carbon dioxide released during respiration and toxic organic solvent vapor released from interior decoration. In order to improve the indoor air quality (IAQ), we must allow outer fresh air into the indoor space and release the dirty air out. But while taking in fresh air, the heat and factory/vehicle exhaust are also introduced. Indoor CO_2_, HCHO, and VOCs and outer dirty gas threaten human health badly. To solve this problem, we bring up an innovative low-power-consuming full-outer-air-intake natural air-conditioning system that completely separates intake and exhaust air, which is a solution for cross-contamination and makes mass/energy exchange by means of air and water. Design airflow exceeds 300∼500 CFM, steam evaporation mass rate reaches 3.13∼3.88 kg/hr, and heat exchange capacity becomes 1,855∼2,300 kcal/hr. The sensible heat effectiveness is 71%∼112%, and EER exceeds 14.05∼17.42 kcal/W·h. In addition, the system under design can be of positive or negative pressure status according to the user's or work's requirement. It creates a comfortable and healthy living environment by supplying clean and fresh outer ambient air with low power consumption.

## 1. Introduction

Nowadays, smog, PM_2.5_, CO_2_, and other air pollutants produced from human activities, progress of technologies, and flue gas and heat emitted from factories and vehicles damage the environment and ecology, creating global warming, climate change, and extreme climate molding. Climate disasters occur worldwide.

Environmental awareness is forced to be focused. Countries all over the world have set up environmental organizations and signed conventions to prevent human beings from continuing to undermine the Earth's ecological environment; for example, COP 21 was the first global agreement in history, used to prevent climate change, signed at the Paris Climate Summit on December 12, 2015, and Paris Climate Change Agreement was signed on April 22, 2016 (called the World Climate Day), at the UN Headquarters in New York, and it came into force on November 4, 2016.

A person stays indoors for about 85%∼90% time of his lifetime, and the need for a comfortable indoor environment is getting higher; thus, the air-conditioning dependency becomes intense too. Nowadays, human beings not only require a comfortable environment but also want the indoor environment to be a comfortable and healthy one.

A closed environment will become hazardous because of carbon dioxide released during respiration and toxic organic solvent vapor released from interior decoration. In order to improve the indoor air quality, we must allow outer fresh air into the indoor space and release the dirty air out. But while taking in fresh air, the heat and factory/vehicle exhaust are also introduced. Indoor CO_2_, HCHO, and VOCs and outer dirty gas threaten human health badly.

Air-conditioning provides a comfortable environment for people; meanwhile, it releases warm and polluted air that damages the Earth's environment seriously. Stopping climate change has become an urgent issue; it is a common responsibility of pursuing the well-being of mankind and establishing a sustainable global village.

### 1.1. Environment inside and outside the Living Space

#### 1.1.1. Environment outside the Living Space

Although air-conditioning evokes scientific and technological progress, economic prosperity, and comfortable environment for mankind, its huge energy consumption and waste heat emissions also bring the Earth an unprecedented catastrophe. The world has been promoting sustainable development for many years; at the same time, there are many “hot islands” in cities, and some are still developing, which is worrisome. Each building discharges a considerable amount of waste heat. Taking Taiwan business buildings as an example, the annual energy consumption per unit area of a business building is about 290 kWh/(m^2^·yr); heat emitted is about 602,040 kcal/(m^2^·yr), which is contrary to the idea of “green buildings” and energy saving.

Thermal emissions of buildings are a very important problem. They impact the ecological environment and even the climate seriously [1]. The heat generated by the building forms the building's heat-island effect, and together with the emissions from industries and traffic waste heat and exhausts, it leads to the urban heat-island effect [2], making the overall building environment temperature rise up to 8–10°C. When the outdoor ambient temperature increases 1°C, the energy consumption increases 6% and energy consumption of air-conditioning operation will increase 48%∼60%, which brings about the superposition of direct thermal emissions [1].

Suspended particles (PM_10_ and PM_2.5_) and chemical gases discharged from vehicles or industrial plants are closely related to the respiratory system and cardiovascular chronic diseases of the human body. No matter how long or short the human body is exposed to the suspended particles (PM_10_ and PM_2.5_), it will cause the insufficiency of cardiac contractility and vascular elasticity, which might become an important factor that causes damage to the cardiovascular system [3].

#### 1.1.2. Living Space Environment

In recent years, changes in the public lifestyle make people enjoy the comfort of the air-conditioning system in a closed living space or office space, which results in “sick building syndrome” (SBS). In a closed building, if the indoor ventilation is poor, the pollutants can easily accumulate and lead to deterioration of indoor air quality.

Indoor air quality is particularly important for children, pregnant women, the elderly, and chronic patients who often stay indoors. This is because children are growing, the proportion of breathing of a child is 50% higher than that of an adult, and 80% time of children's lifetime is spent indoors. Children are more susceptible to air pollution than adults [4, 5]. The WHO's research report pointed out that people died from indoor air pollution and asthma are around 10,000 worldwide every year, of which 35% are children; therefore, the indoor air quality has become an international topic and cannot be ignored.

The presence of air pollutants is a serious threat to human health, especially to the human respiratory system and the cardiovascular system [6].

### 1.2. Main Factors That Affect the Comfort of Human Beings

“Comfortable environment” is an ideal type of surrounding that people have long pursued. With the industrial revolution, the quality of human life has been greatly improved by leaps. In spite of enhancing the comfort of the indoor environment, it has also caused environmental pollution. The proportion of time Americans stay indoors is about 89% per day, and the remaining 11% time is spent in vehicles and outdoors [7]. With the technological progress and urbanization, time being spent indoors is increasing too.

The main factors affecting human comfort are the indoor temperature, relative humidity (RH), wind velocity, and noise. Nowadays, the quality of human life has improved; with the issue of indoor air quality, “sick building syndrome (SBS)” has also become an important focus. The impact factors are carbon dioxide, volatile organic compounds, biological aerosols, and so on. These substances recirculating in the building become the largest culprit in degrading indoor air quality [8]. The World Health Organization (WHO) has also proposed various concentrations for different pollutants such as SO_2_, NO_2_, CO, and ozone and studied about the relation of impacts on human health or increasing possibility of illness to long-term and short-term exposure to them [9].

According to the ASHRAE 55-2013 specification [10], the metabolic rate of the human body is 1.2 Met (1 Met = 58.15 W/m^2^); with regard to typical clothing conditions, the summer comfort temperature in Taiwan is 23°C∼26°C and winter comfort temperature is 18°C∼20°C. In the Serra research report, even if the temperature is higher or lower, the fluctuation of relative humidity affects the human body's heat balance and warm feeling [11]. In the Taiwan region, 50% to 60% RH and 0.15∼0.3 m/sec per minute airflow distribution are required. In general, higher airflow can easily cause human discomfort. In the indoor air-conditioning environment, the more gentle the airflow is, the more comfortable the human body feels. Indoor air cleanliness determines the indoor air quality (IAQ). According to a study conducted by the American Building Owners and Management Association (BOMA), each person in the room requires at least 20 CFM (cubic feet minute).

### 1.3. Minimum Energy Consumption Can Improve Air Quality

Closed buildings with insufficient fresh airflow result have poor air quality in the indoor environment and enormous organic chemical pollutants. Therefore, a comfortable environment does not represent a healthy one. To improve the indoor air quality, buildings most commonly use natural or mechanical ventilation systems. But using mechanical ventilation systems to take in outer fresh air and improve the indoor air quality will produce a lot of heat and consume considerable energy from motor running.

Nowadays, they rather promote energy conservation worldwide. While running the building's ventilation system, energy consumption can be reduced by monitoring indoor CO_2_ concentration from the CO_2_ sensor, which targets to control the CO_2_ concentration value at a set level and reduce power waste from the ventilation system. In the studies [12, 13], the indoor air quality has been accurately controlled by building a CO_2_ concentration prediction model and reducing energy waste from the ventilation system. In studies [14–17], indoor CO_2_ concentration has been detected through the CO_2_ sensor and more accurate control of indoor air quality has been achieved through the built CO_2_ concentration prediction model, while achieving energy-efficient buildings. Having the ventilation system accommodate the throttling valve or others can limit the air-intake amount and achieve the energy-saving purpose [18].

In the aforesaid energy conservation study on the building ventilation system, different plans have all achieved good results, but these plans have not considered the fact that fresh air intake also bring in the air pollutants and heat that increase the building air-conditioning load and energy waste; energy consumption of an air-conditioning system is more than that of a ventilation system in a building; therefore, while sucking in outdoor air, it also should consider the incurred heat load and must consider both the air-conditioning systems and ventilation systems at the same time; the energy conservation potential is remarkable.

The motivation of this study is to seek a thorough solution to the problem of reduced indoor air quality and massive increase of energy consumption by the air-conditioning system from sucking in outdoor air, design a way to allow fresh air from outdoors, improve indoor air quality, and reduce the external air heat load with the minimum energy consumption; meanwhile, the suspended particulates (PM_10_ and PM_2.5_) in the air should be captured by means of an innovative low-energy-consuming full-outer-air-intake natural air-conditioning system. The main purpose is to build a prototype of healthy environment and energy-saving air-conditioning system, achieve the target of improved air quality in buildings with lowest energy consumption, and ensure not to pollute or jeopardize the environment.

This article's structure is illustrated as follows: Section 2 interprets the architecture of innovative low-energy-consuming full-outer-air-intake natural air-conditioning system.  Section 3 presents experimental configuration and performance testing and comparisons with the presented innovative systems.  Section 4 concludes the study.

## 2. Architecture of Innovative Low-Energy-Consuming Full-Outer-Air-Intake Natural Air-Conditioning System

As mentioned above, this study aims to achieve the minimum energy consumption for air-conditioning systems and create a natural healthy environment. We illustrate the architecture, characteristics, and advantages and disadvantages of the traditional mechanical ventilation system and propose the innovative low-energy-consuming full-outer-air-intake natural air-conditioning system; that is, we use an innovative building energy conversion system and completely improve the problems of the traditional system. This section describes the architecture of the planned system.

### 2.1. Traditional System Architecture

Today's buildings mostly apply energy conversion systems as the total heat exchanger; the conventional total heat exchangers are divided into two types: the “rotary wheel” type and the “polymer fixed plate” type.

#### 2.1.1. Rotary Wheel Type

**Figure 1:**
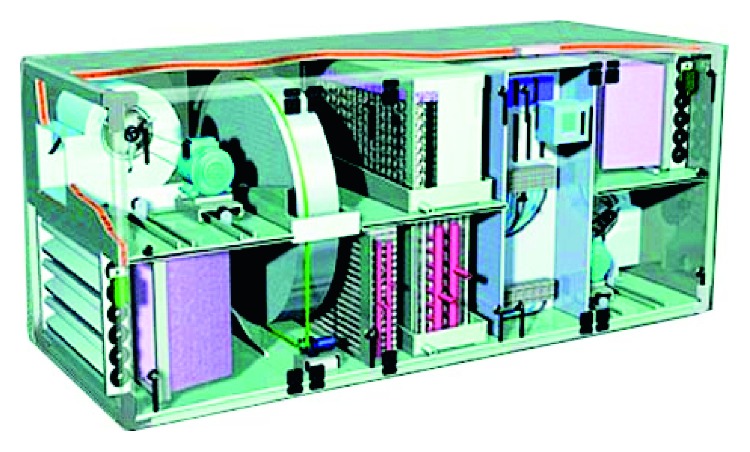
Heat exchanger wheels [19].

**Figure 2:**
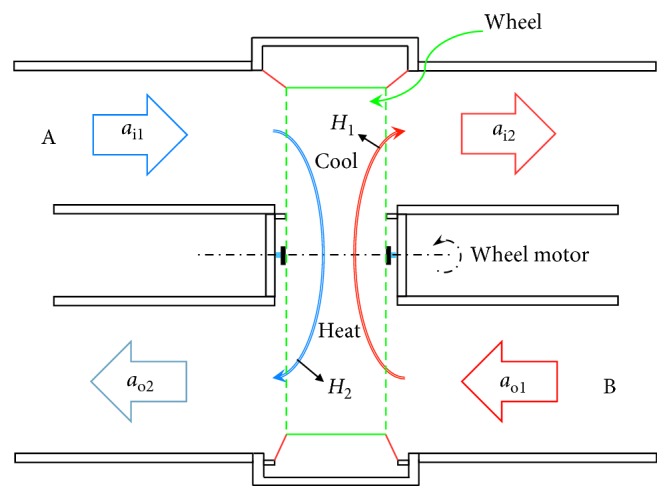
Conventional total heat exchanger structure demo diagram.

The rotary wheel-type heat exchanger is shown in Figure 1 [19]. It uses the motor to drive the honeycomb wheel; the honeycomb contains numerous parallel channels to increase the heat exchange area. The wheel can be divided into two sides: indoor air duct (A) and fresh airway (B), as shown in Figure 2. When the sucked outdoor air (*a*_o1_) passes through the hot rotary wheel, the wheel absorbs heat, the sucked outer air temperature (*H*_1_) decreases, and the cooled fresh air (*a*_o2_) is supplied to the room; the saturated part is continuously transferred to the other side, where indoor air duct discharges the indoor cold air (*a*_i1_) with lower temperature and humidity, which is the wheel heat discharge. The discharged air temperature rises up (*H*_2_) so that the temperature of discharged indoor air (*a*_i2_) is closer to the outdoor temperature, thus achieving the cooling and dehumidifying effect.

- Advantages of this arrangement are that the heat exchange efficiency elevates to about 65%∼78% and it is suitable for large systems or collectedly treated systems on outside air, such as the outer-air-conditioning box used for the central air-conditioning system.
- The shortcoming is that, since the duct applies mixing of intake and discharge air, cross air pollution problem occurs on intake/discharge air, and thus, there is a need for an auxiliary heat source to dehumidify the wet air.

#### 2.1.2. Polymer Fixed Plate

**Figure 3:**
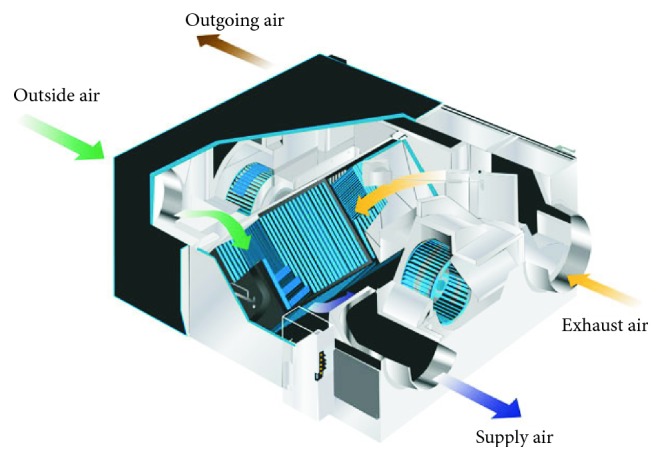
Polymer fixed plate energy recovery [20].

**Figure 4:**
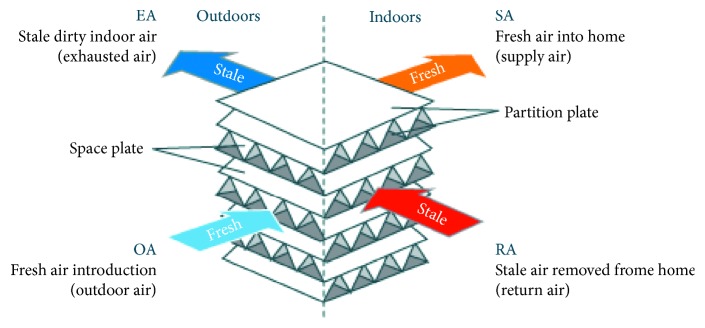
Polymer fixed plate [21].

The polymer fixed plate-type heat exchanger is shown in Figure 3 [20]. There are many plates in the heat exchanger that build the air runner and are separated to two flow passages on each side of each plate with the partitions and the sealing device; the flow directions are crossover, as shown in Figure 4 [21]. The heat exchange structure material is made of highly permeable fibers; moisture from the outer air at high temperatures is allowed to penetrate the air with low temperature/humidity at the other side and is discharged outdoors in this way to achieve heat exchange benefit.

- The advantages are that the heat exchange efficiency elevates about 65%∼78%, it is suitable for homes, offices, and other small- and medium-spaced buildings, and it has easy maintenance.
- The disadvantage is that intake and discharge channels are separated, but the heat exchange material uses high permeability fibers and will still contaminate the fresh intake air by the discharged air.

The total heat exchangers have improved the air quality in the building, but the outdoor air heat they introduce will still increase the load of air-conditioning systems, and the air cross-contamination problem still exists.

### 2.2. Planned Innovation System Architecture

Current buildings use the total heat exchangers to carry out internal and external ventilation, resulting in elevation of air-conditioning load and air cross-contamination issues. The components of this innovative building energy conversion system can be divided into the water evaporation plant, heat pipes, cold water heat exchanger, VAV fan, pump, control master, etc.

The most important feature of this innovative system is to combine the water evaporation plant and the heat pipes, allow fresh air into the building, and discharge indoor dirty air; the building's inside/outside energy recovery and energy balance can avoid the external high-temperature and high-humidity air intruding into the indoor space and prevent indoor cold energy from being discharged to outside. Since not using other heat-source power, the cold energy recovery is driven by gravity so that the movable components of the innovative system are the fan and pump motors only; the use of compressor power can be abandoned to reduce the power consumption, with the features of energy saving, indoor air quality improvement, deduction of waste heat emission, environmental protection, etc.

**Figure 5:**
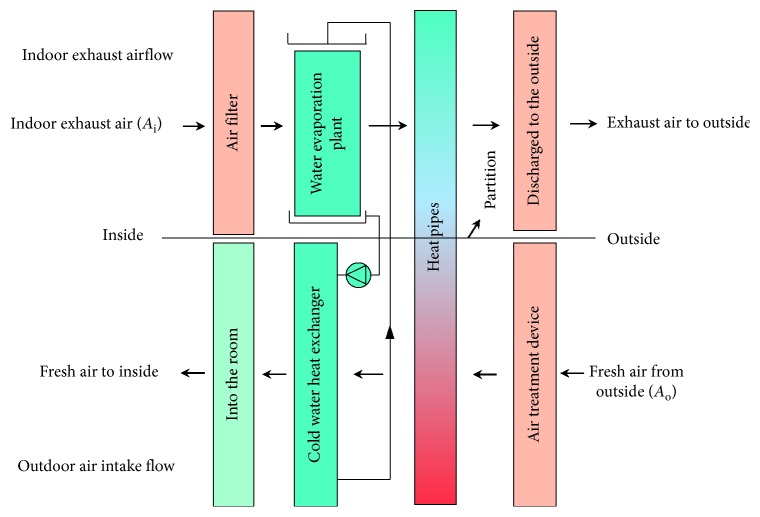
Architecture of the innovative energy conversion system for building ventilation.

This study first explores the system architecture on the situation that both the indoor air temperature and humidity are lower than the outdoor air temperature and humidity, as shown in Figure 5. Airflow paths can be divided into two parts: indoor exhaust flow and outdoor air intake flow. First, for the indoor exhaust flow (*A*_i_), since the indoor air temperature and humidity are lower than those of outdoor air, the exhaust passes through the water evaporation plant, gets cooled by means of water evaporation to absorb heat so that indoor exhaust flow turns to high-humidity saturated air, discharges its heat through the heat pipes and becomes the high-temperature air with the same humidity, and then discharges to the atmosphere.

On the contrary, for the outdoor air intake flow (*A*_o_), high-temperature outdoor air flows through the evaporation section of heat pipes reducing its enthalpy and gets cooled by the water heat exchanger, further reducing air enthalpy and becoming the fresh air being cooled and dehumidified with two processes and sent into the house.

VAV fan is applied in both the indoor exhaust and outdoor intake processes, and different control modes can be used under different circumstances.

#### 2.2.1. Energy Conversion Is Carried Out by Steam Partial Pressure in the Air

Air and water are natural energy carriers. Water vaporization and condensation are natural patterns of energy transfer. The water evaporation plant uses the difference of water vapor molecules between the building's indoor air and outdoor air that creates the steam partial pressure and makes energy heat transfer; when the steam partial pressure between building's indoor air and outdoor air is high, the absolute humidity difference is also high, and from the fact that water can filter and purify air, it will capture the particulate matter during heat exchange; in this innovative system, air intake and exhaust channels are separated from each other completely, which effectively avoids the cross-contamination problem during the air exchange process.

**Figure 6:**
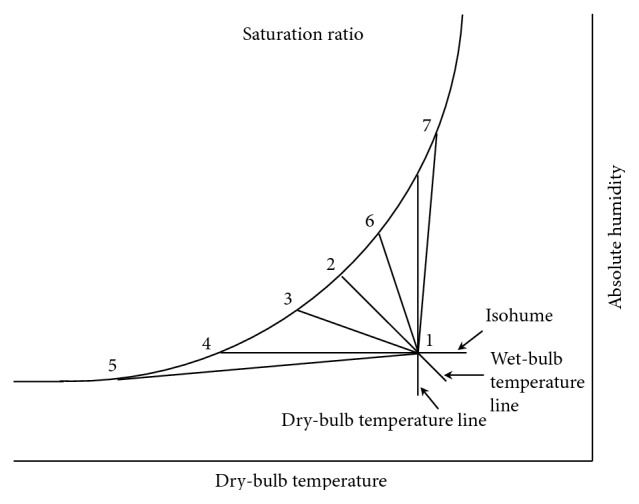
Energy conversion chart of steam partial pressure in the air.

The modulating processes of various water evaporation plants on psychrometrics are shown in Figure 6; the dry-bulb temperatures in all processes must proceed toward the saturated humidity line, allowing the air to reach or approach its saturation humidity state.

The process of line 1 ⟶ 2 shown in Figure 6 is called evaporative cooling process. Air flows through the water evaporation plant, and heat in water does not increase nor decrease; excluding the pump and other external factors, while air is flowing through the water evaporation plant, the wet-bulb temperature line moves toward the saturated humidity line and remains at that specific temperature.

If the water is subjected to be properly cooled before coming into the water evaporation plant to contact with air, the slope of process line moves down the natural evaporative cooling line, as shown in line 1 ⟶ 3 in Figure 6. The partial cooling leads to lower dry-bulb and wet-bulb temperatures of leaving air, but when the moisture content is higher than that of the entering air, this process is called the cooling and humidifying process.

If the water is cooled again, it will make sensible cooling; this process is shown as the 1 ⟶ 4 line in Figure 6. The dew point of incoming air is exactly equal to the effective surface temperature of the water evaporation plant. When the air leaves the water evaporation plant, lower dry-bulb and wet-bulb temperatures are obtained, but the moisture content is the same as that of the incoming air. This process is called the sensible cooling process.

When the water temperature is much lower than the dew point of incoming air, the dry wet-bulb temperature and moisture content will be reduced after the air comes into contact with water, which makes the cooling and dehumidifying effect. This process is called the cooling and dehumidifying process, shown as line 1 ⟶ 5 in Figure 6.

When the water is heated to the appropriate temperature and mixes with air in the water evaporation plant, but the heat is not enough to make the air temperature rise, then the leaving air only lowers its dry-bulb temperature and both the wet-bulb temperature and moisture content are higher than those of the incoming air; this process is called the cooling and humidifying process, shown as line 1 ⟶ 6 in Figure 6.

If the water takes sufficient heat from heating and then goes through the water evaporation plant, the process is shown as the 1 ⟶ 7 line in Figure 6, and the dry-bulb temperature, wet-bulb temperature, and moisture content of the air leaving the water evaporation plant are higher than those of the incoming air; this process is called the heating and humidifying process (change of steam spray).

#### 2.2.2. Heat Pipes Perform Energy Conversion

**Figure 7:**
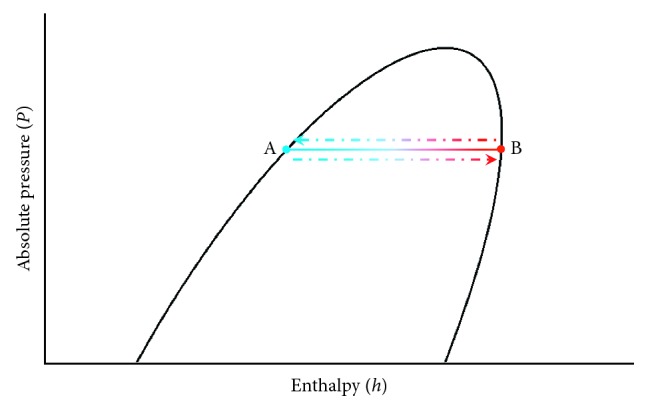
Mollier diagram of energy conversion of heat pipes.

The heat pipes use the liquid-gas phase change to make heat transfer, and the heat transferred is large and rapid, making them suitable as an energy conversion device.  Figure 7 shows that when the high-temperature outdoor air goes through the heat pipes, the process A ⟶ B is the heat-absorbing evaporation process and air temperature is relatively lower than that of the incoming one; when the low-temperature air goes through the heat pipes, the process B ⟶ A is the heat-discharge condensation process and air temperature is relatively higher than the intake temperature.

**Figure 8:**
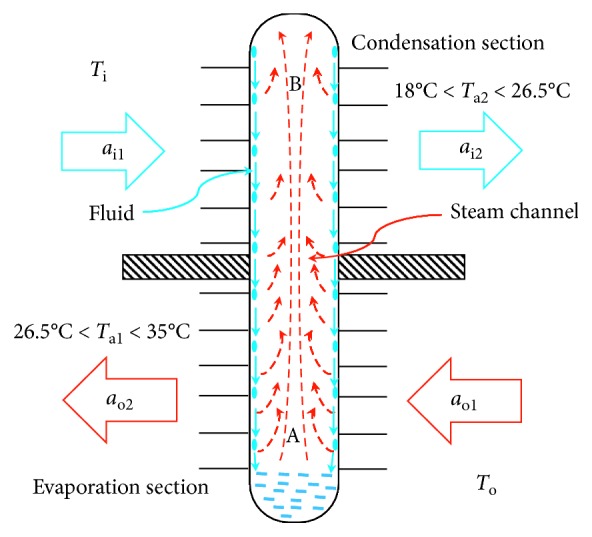
Demo diagram of the liquid-gas phase change in heat pipes.

Heat pipes have very high thermal conductivity; heat pipes have the advantages of no additional auxiliary heat source power required, light weight, rapid response to the change of heat load, etc. Heat pipes can be divided into the following sections, as shown in Figure 8:

1. Heat-release condensation section (B side): when the gaseous working fluid condenses into liquid and releases heat, it is the heat-release section of heat pipes; the condensed liquid returns to the evaporation section by gravity.
2. Evaporation section (A side): when the condensed working fluid is evaporated herein, it is the cooling section of heat pipes; the evaporated gaseous fluid naturally drifts up to the condensation section by means of thermal buoyancy.
3. Steam channel: the gaseous working fluid produced in the evaporation section must have a flow space, allowing it to flow smoothly into the condensation section.

Working fluid uses the condensation and evaporation characteristics and makes the heat pipes have the cooling and exothermic capability.

If indoor temperature (*T*_i_) = 18°C and outdoor temperature (*T*_o_) = 35°C, while the incoming air (*a*_o1_) goes through A side of heat pipes making heat exchange, temperature (*T*_*a*1_) of air (*a*_o2_) coming to indoor space is within 26.5°C∼35°C; when the indoor air (*a*_i1_) discharges out, it goes through B side of heat pipes making heat exchange and temperature (*T*_*a*2_) of air (*a*_i2_) discharging out is within 18°C∼26.5°C.

Based on the aforesaid water molecular pressure difference and heat pipe principle, we can achieve the proposed energy conversion system.

## 3. Experimental Configuration and Testing on the Innovative Building Energy Conversion System

### 3.1. Operating Theory Analysis

**Figure 9:**
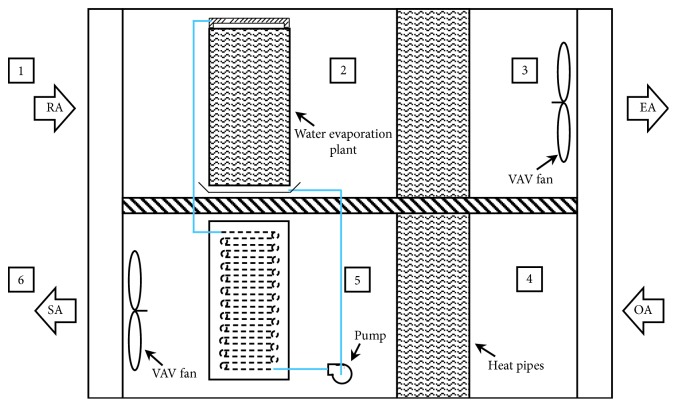
Building energy conversion system prototype configuration and temperature and humidity measuring points.

Based on the principle and framework of the innovative building energy conversion system, we have designed the prototype of it and carried out the running performance test in the prospective of realizing the operation status of the building energy conversion system (Figure 9). This study will discuss the property changes of indoor air and outdoor air after passing through the building energy conversion system to make energy conversion.

**Figure 10:**
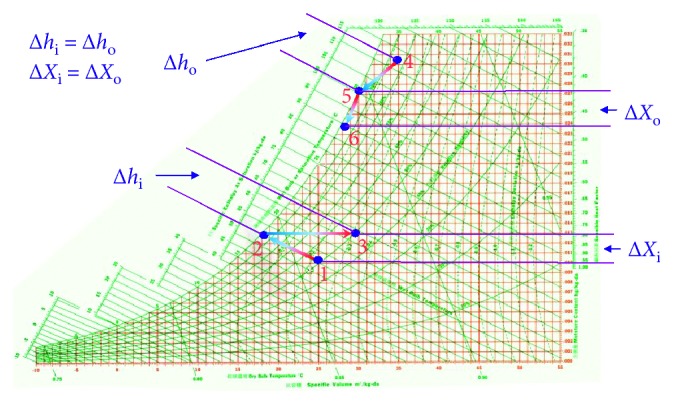
Graph showing building air-circulation theory.

The building energy conversion system provides the energy balance between the inside and the outside of the building, uses 25°C temperature and 50% relative humidity as the data, sucking outdoor fresh air at 25°C∼35°C and 40%∼90% relative humidity state into the enclosed indoor space, and analyzes the changes by psychrometrics, as shown in Figure 10.

The air-discharge energy conversion process is state 1 ⟶ state 2 ⟶ state 3.

State 1 is the initial known state of the indoor environment (temperature and humidity); other air properties are obtained by psychrometrics (Figure 10).

State 2 is the state in which indoor exhaust goes through the water evaporation plant making adiabatic evaporation; some water absorbs heat from air and evaporates and mixes up with air, making the water evaporation plant temperature drop to the wet-bulb temperature of discharged air and recovery of the air-conditioning cold energy; after passing through the water evaporation plant, indoor exhaust also gains the moisture, and its temperature is reduced to its own wet-bulb temperature, as shown in Figure 10.

After going through the water evaporation plant at state 2, the indoor exhaust air becomes low-temperature saturated air and then is discharged to atmosphere through the heat pipes, which are at state 3 shown in Figure 10.

The energy conversion process of outdoor incoming fresh air is state 4 ⟶ state 5 ⟶ state 6.

State 4 is the known state of the outdoor environment with ambient temperature and humidity; other air properties by psychrometrics are obtained (Figure 10).

After state 2, incoming fresh air goes through state 5 in which it passes through the air filter device where the impurities in the air are filtered out and then through the heat pipes where the heat of high-temperature air sucked is transferred to the heat pipes; this process depends on the temperature of internal fluid inside the heat pipes and gets the cooling-down effect, as shown in Figure 10.

After going through the heat pipes at state 5, the fresh air presents the low-temperature and low-humidity state, and finally, it goes through the cold water heat exchange tubes to cool and dehumidify once again; this process depends on the temperature of cold water in heat exchange tubes. After two cooling and dehumidifying processes, the fresh air is sent into the room at state 6 (Figure 10).

#### 3.1.1. Indoor Exhaust

When air enthalpy at state 2 changes to state 3, the indoor exhaust air changes the enthalpy in the value of enthalpy difference (∆*h*_i_) expressed by equation (1); the state-change process from state 1 to state 3 will produce an absolute humidity difference (∆*X*_i_), expressed by equation (2). The enthalpy difference (∆*h*_i_) and absolute humidity difference (∆*X*_i_) represent how much energy in the indoor exhaust air is converted.

#### 3.1.2. Fresh Air Intake

When air enthalpy changes from state 4 to state 5, the fresh air intake changes the enthalpy in the value of enthalpy difference (∆*h*_o_) expressed by equation (3); the state-change process from state 5 to state 6 will produce an absolute humidity difference (∆*X*_o_), expressed by equation (4). The enthalpy difference (∆*h*_o_) and absolute humidity difference (∆*X*_o_) represent how much energy in the fresh air intake is converted:

(1) $$\begin{array}{c} \Delta h_{\text{i}}=\left|h_{\text{i}1}-h_{\text{i}2}\right|, \end{array}$$

(2) $$\begin{array}{c} \Delta X_{\text{i}}=\left|X_{\text{i}1}-X_{\text{i}2}\right|, \end{array}$$

(3) $$\begin{array}{c} \Delta h_{\text{o}}=\left|h_{\text{o}1}-h_{\text{o}2}\right|, \end{array}$$

(4) $$\begin{array}{c} \Delta X_{\text{o}}=\left|X_{\text{o}1}-X_{\text{o}2}\right|, \end{array}$$

where *h*_i*n*_ is the indoor exhaust air enthalpy, *X*_i*n*_ is the indoor exhaust air absolute humidity, *h*_o*n*_ is the fresh incoming air enthalpy, *X*_o*n*_ is the fresh incoming air absolute humidity, ∆*h*_i_ is the indoor exhaust air enthalpy difference, ∆*X*_i_ is the indoor exhaust air absolute humidity difference, ∆*h*_o_ is the incoming fresh air enthalpy difference, and ∆*X*_o_ is the incoming fresh air absolute humidity difference.

Following 2000 ASHRAE Handbook [22], the outdoor ambient temperature (OA_*T*_), indoor ambient temperature (IA_*T*_), and intake air temperature (SA_*T*_) in the building energy conversion system are measured, and the sensible heat exchange efficiency (*η*_s_) of energy conversion is evaluated as follows:

(5) $$\begin{array}{c} \eta_{\text{s}}=\frac{{\text{OA}}_{T}-{\text{SA}}_{T}}{{\text{OA}}_{T}-{\text{IA}}_{T}}\times 100\%. \end{array}$$

Following 2004 ASHRAE Handbook [23], the air enthalpy difference in the building energy conversion (∆*h*) and air mass (*m*) are measured, and the refrigerating capacity (*Q*) is evaluated as follows:

(6) $$\begin{array}{c} Q=m\left(h_{1}-h_{2}\right). \end{array}$$

The refrigerating capacity (*Q*) is calculated, the rated consumed power (*W*) is found, and the energy efficiency ratio (EER) of the building energy conversion system is evaluated, as follows:

(7) $$\begin{array}{c} \text{EER}=\frac{Q}{W}. \end{array}$$

### 3.2. Experimental Environment Arrangement

**Figure 11:**
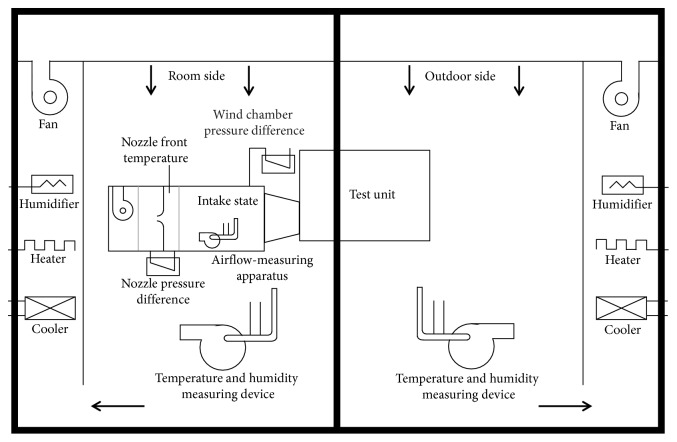
Experimental environment of the building energy conversion system.

**Figure 12:**
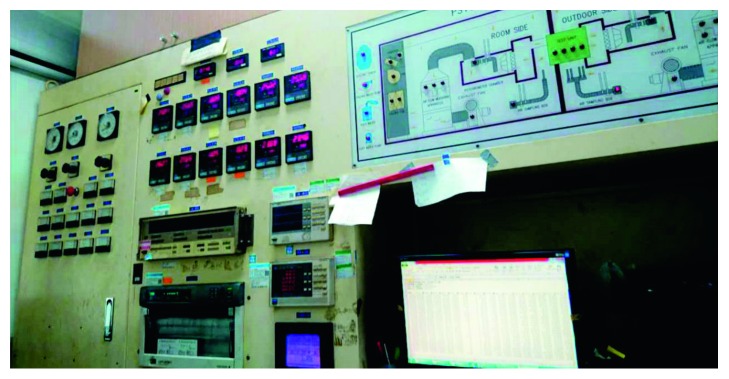
Lab environment control instruments and recording system.

The VAV fan prototype of the building energy conversion system is 300∼500 CFM; the experimental environment of this study is shown in Figures 11 and 12. The experimental environment arrangement and indoor environmental condition of the building energy conversion system are shown in Table 1.

Outdoor environmental conditions in the building energy conversion system test are dry-bulb temperature = 35°C and RH = 80%∼45%.

### 3.3. Performance Verification and Efficiency Comparison

The indoor/outdoor lab conditions of the building energy conversion system are as follows: indoor: dry-bulb temp. = 25°C and relative humidity (RH) = 50%; outdoor: RH is adjusted to 80% ∼ 45% taking experiments; the outdoor environmental condition is recorded once per each 5% change of RH, and the dry-bulb temperature, RH, and absolute humidity of incoming fresh air are also recorded; 8 points are recorded in total.

**Figure 13:**
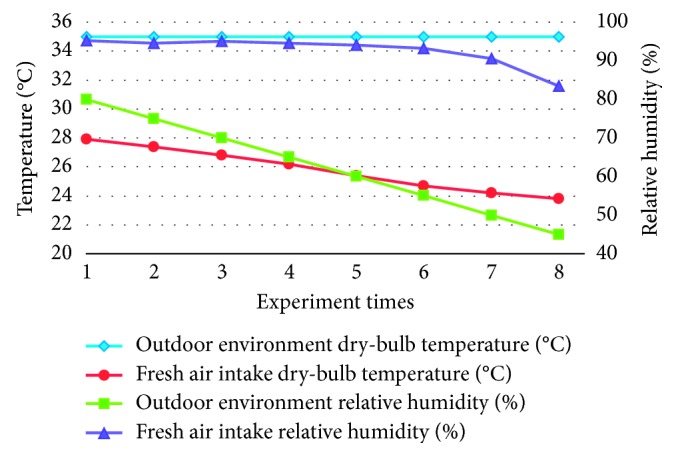
Experimental data curve of the air intake states under different RH conditions.

In addition, the fresh air temperature and relative humidity changes that are introduced into the room through the proposed energy conversion system are measured under different outdoor humidity conditions. When the external air dry-bulb temperature is at 35°C, the temperature change of the introduced fresh air is measured under different outdoor relative humidity conditions from 80 to 45%. After the energy conversion, the dry bulb shows that the temperature of the fresh air introduced can be reduced to 27.9°C∼23.8°C, respectively, as shown in Figure 13.

**Figure 14:**
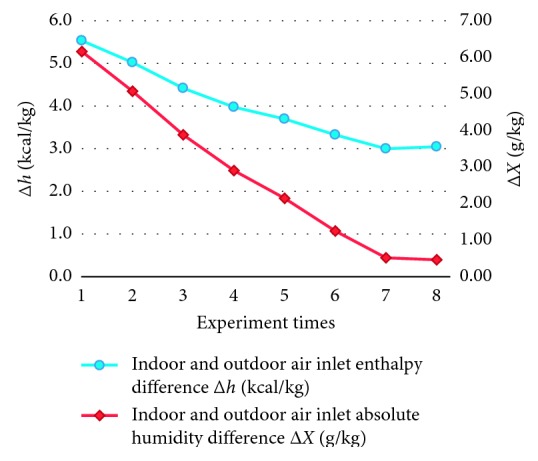
Air intake/exhaust: air enthalpy difference and absolute humidity difference curves.

The analyzed resultant data of air properties from indoor air exhaust and outdoor air intake in the experiment are shown in Figure 14. From Figure 14, it can be seen that the enthalpy difference between the air intake and air exhaust is ∆*h*_i_ = ∆*h*_o_ = 2.99∼5.54 kcal/kg and absolute humidity difference is ∆*X*_i_ = ∆*X*_o_ = 0.45∼6.15 g/kg.

**Figure 15:**
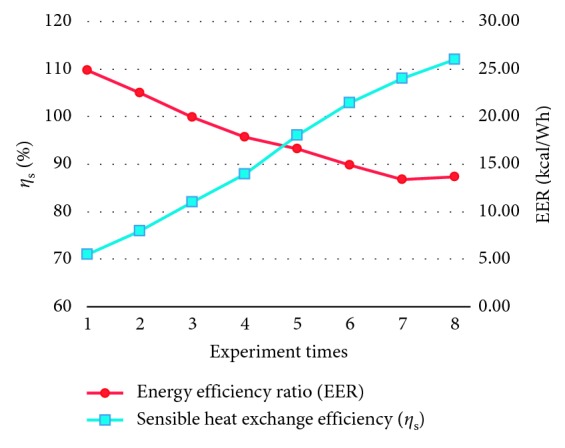
Sensible heat exchange efficiency and energy efficiency ratio curves.

The analyzed resultant data of sensible energy conversion efficiency (*η*_s_) and energy efficiency ratio (EER) are shown in Figure 15. From Figure 15, it can be seen that the *η*_s_ is within 71%∼112% and EER is within 24.87∼13.69 kcal/W·h.

While the building energy conversion system is making air intake/exhaust exchange, it makes heat exchange by means of the steam partial pressure between air and water evaporation plant. Since the wet-bulb temperature of incoming air varies, the evaporating rate is within 5.54∼2.99 kg/hr, the heat exchange capacity is 3283∼1772 kcal/hr, sensible energy conversion efficiency (*η*_s_) is 71%∼112%, and the energy efficiency ratio (EER) is 24.87∼13.69 kcal/W·h. From this, it can be seen that the RH of incoming air will affect the heat exchange capacity of the subjected innovative system; but it can still achieve the benefits of air cooling and dehumidification.

**Figure 16:**
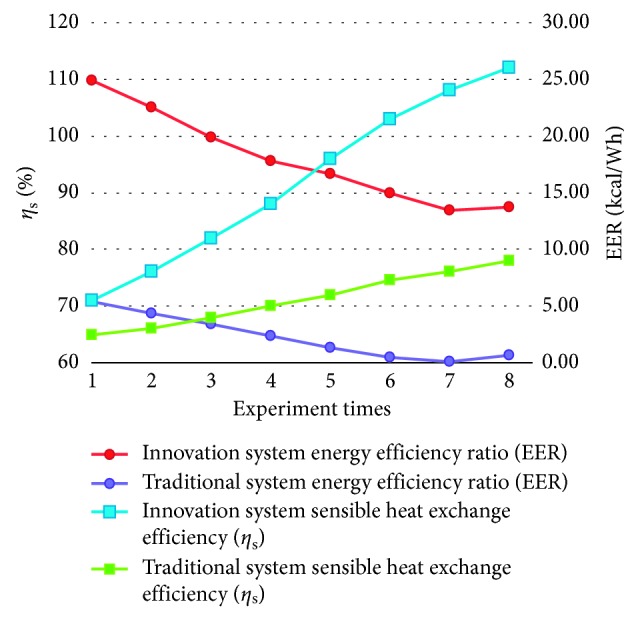
Sensible heat exchange efficiency (*η*_s_) and energy efficiency ratio (EER) curves for traditional and innovative systems.

The sensible energy conversion efficiency (*η*_s_) and energy efficiency ratio (EER) are often used to evaluate the merits of the building ventilation system. In this study, sensible energy conversion efficiency (*η*_s_) and energy efficiency ratio (EER) were used to evaluate and compare the performances of the designed system and the traditional full heat exchange system. The relevant test results are shown in Figure 16. From Figure 16, it can be seen that, under the same environmental condition, the sensible energy conversion efficiency (*η*_s_) is 65%∼78% and the energy efficiency ratio is 5.43∼0.07 kcal/W·h for the traditional total heat exchanger system.

Compared with the traditional total heat exchanger system, the subjected innovative system improves the sensible energy conversion efficiency (*η*_s_) by 6%∼34% and energy efficiency ratio (EER) performance by 19.44∼13.62 kcal/W·h. The overall performance is better than that of the conventional total heat exchanger system.

**Figure 17:**
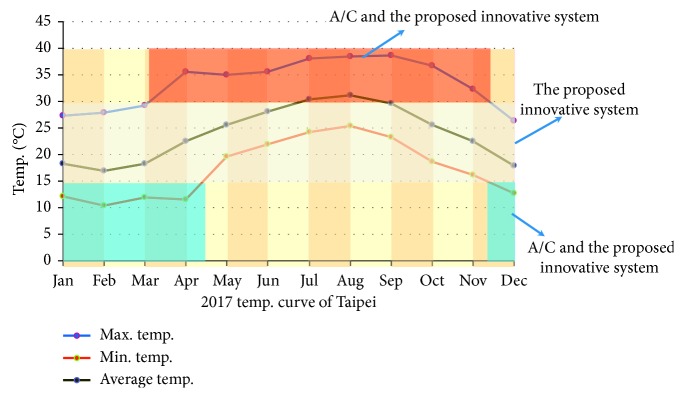
The operation feature map of the proposed energy conversion system.

Figure 17 shows the annual temperature distribution of Taipei City in 2017. In Figure 17, the red curve indicates the lowest temperature of each month, while the blue and the dark green curves correspond to the highest and the average temperature of each month, respectively. As can be seen in Figure 17, the temperature variation between day and night in March, April, October, and November is large in Taipei City. The maximum temperature during the day can be as high as 36°C, while the nighttime temperature can be as low as 15°C or less. When the external air temperature is within 15°C∼30°C, the proposed energy conversion system has the ability of natural cooling and a large amount of external air can be introduced without the need to start the air-conditioning system. The external air temperature can be reduced about 3°C∼5°C so that a comfortable environment can be obtained. When the external air temperature is higher than 30°C (the red block region in Figure 17) or less than 15°C (the blue block region in Figure 17), the air-conditioning system needs to be activated to provide cool or hot air to maintain a comfortable indoor environment. In fact, as we mentioned in Section 3.3, when the outdoor air temperature is high and the relative humidity is low, the proposed system will achieve better natural cooling performance. Consider Figure 13 as an example. When the outdoor air temperature is 35°C and the relative humidity is 45%, the external air temperature can be reduced to 23.8°C via energy conversion technique of the proposed system. Therefore, the proposed system can effectively reduce the temperature of the introduced indoor air under low energy consumption conditions to create a comfortable environment.

**Figure 18:**
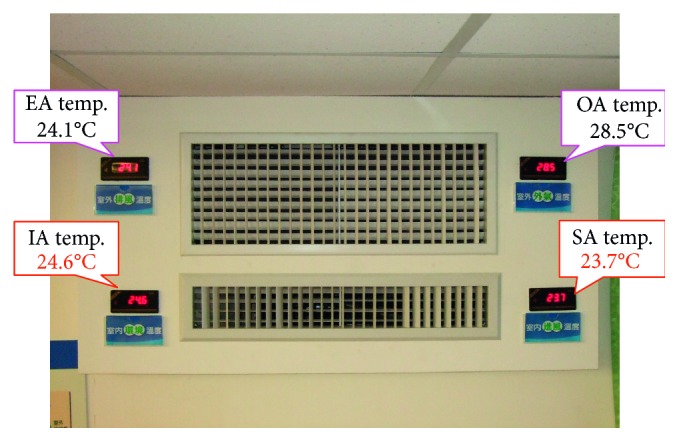
The proposed energy conversion system in the test site (classroom).

In addition, we use a 1500 CFM building energy conversion system in a classroom with an area of 80.75 m^2^ and a capacity of 30 students in one of the universities in Taiwan as a verification field.  Figure 18 shows the system we have set up. As can be seen in Figure 18, the introduced outdoor air (OA) temperature is 28.5°C; because the outdoor air temperature is between 15°C and 30°C, there is no need to turn on the air-conditioning system, and the external air will be introduced directly. After the introduction of the external air through the system, the intake air (SA) temperature is 23.7°C; that is, the outdoor air temperature is reduced by 4.8°C via the proposed system, and the introduced external air temperature is 0.9°C lower than the indoor ambient (IA) temperature of 24.6°C. The sensible energy conversion efficiency (*η*_s_) of the system in the teaching classroom is 123%.

**Figure 19:**
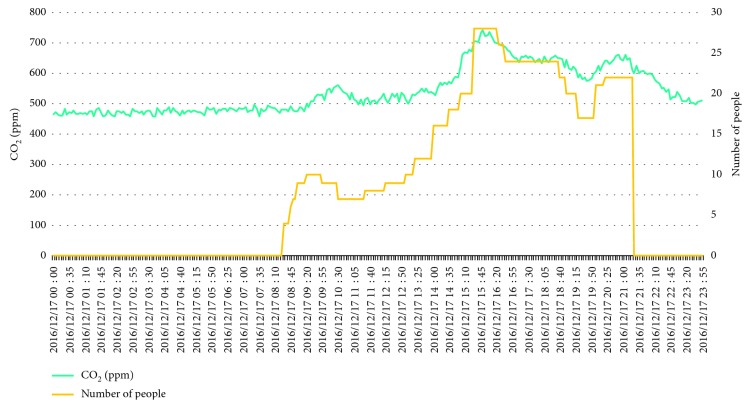
Number of students and corresponding indoor CO_2_ concentration with respect to time in the test site.

Figure 19 shows the relationship between the number of students and corresponding indoor CO_2_ concentration with respect to time in the test environment (i.e., the classroom). As shown in Figure 19, when the number of students in the classroom is 28, the indoor CO_2_ concentration can be up to 736 ppm; the air quality is still in a comfortable condition because the CO_2_ concentration can be maintained below 800 ppm. For the rest of the time, the CO_2_ concentration is between 480 ppm and 650 ppm, so the system can provide a comfortable and healthy environment.

This innovative system solves the problem of air cross-contamination existing in the total air exchanger. Due to the rotary wheel airflow design of the total heat exchanger system, the airflow channels are not separated at the runner so that entering air and exiting air mix up, causing the fresh air to contaminate at the runner. In addition, the polymer fixed plate also has this problem; the partition used to separate the intake/exhaust air in the polymer fixed plate heat exchanger applies fiber materials with high permeability, which will contaminate the incoming fresh air. In the subjected innovation system, air channels are isolated independently, completely avoiding the air cross-contamination between the incoming air and exhaust air. Features of the subjected innovative system are as follows: First, water is used as the energy carrier; when air goes through the water evaporation plant, the energy conversion is made by the steam partial pressure, plus the cold capacity recovery and humidity adjustment are made by heat pipes, enabling the subjected innovative system to get the best heat-exchanging effect. Second, the system can accommodate users' special needs, adjusting the air intake/exhaust amount of the building energy conversion system to present a positive or negative pressure environment. Feature comparisons of this subjected innovative system and traditional total heat exchanger system are listed in Table 2.

## 4. Conclusion

The experiment results show that, for both the sensible energy conversion efficiency (*η*_s_) 71%∼112% and energy efficiency ratio (EER) 24.87∼13.69 kcal/W·h, this subjected innovative system performs better than the traditional total heat exchanger system. The subjected innovative system makes heat exchange, in the most natural way through the air and water energy conversion, and thus can solve the cross-contamination problem occurring in the traditional total heat exchanger. The low-energy-consuming full-outer-air-intake natural air-conditioning system presented in this study article aims to build the best ventilation system; that is, building a low-power-consuming respiratory system, in the absence of pollution, to provide clean air and improve indoor air quality, creates both a comfortable and healthy environment. The design of the system can also be in accordance with environmental conditions and adjust the intake and exhaust airflow rate to create either a positive or negative pressure environment. This system not only has a high degree of energy conversion efficiency but also is applicable to different environments, with a high degree of practicality.

## Figures and Tables

**Table 1:** Indoor environmental condition.

Item	Parameter	Value
1	Dry-bulb temp. (°C)	25
2	Wet-bulb temp. (°C)	17.89
3	Abs. humidity (g/kg)	9.87
4	RH (%)	50
5	Dew point temp. (°C)	13.88
6	Air specific wt. (m^3^/kg)	0.86
7	Air enthalpy (kcal/kg)	12.01
8	Air delivery (ft^3^/min)	500
9	Rated consumed power (W)	132

**Table 2:** Comparison table of the building energy conversion system and traditional total heat exchanger system.

Item	This subjected system	Total heat exchanger	
		Rotary wheel	Polymer fixed plate
Energy conversion method	Air and water	Air	Air
Fan power	Variable air volume	Constant air volume	Constant air volume
Equipment power	Fan and pump	Wheel motor and fan	Fan
Separation of intake/exhaust air	Entirely separated	Mixed up	Separated by highly permeable fibers
Air cross-contamination	No	Yes	Yes
Positive/negative environment	Yes	No	No
Sensible energy conversion efficiency	71%∼112%	65%∼78%	65%∼78%
EER	24.87∼13.69 kcal/W·h	N/A	5.43∼0.07 kcal/W·h

N/A: unable to measure EER because the wheel-type total heat exchanger does not provide a similar model.

## Data Availability

The parameters and test data regarding this research are available at https://drive.google.com/open?id=1KXUI_6EAoHofpj_iyzNJBG9yZfWdw_j.
